# Abdominal Compartment Syndrome Secondary to Constipation in an Adult Patient With Cerebral Palsy

**DOI:** 10.7759/cureus.46312

**Published:** 2023-10-01

**Authors:** Jocelyn Zi Lin Ting, M Priya Dharshini, Mei Fang Chew

**Affiliations:** 1 Emergency Medicine, Tan Tock Seng Hospital, Singapore, SGP; 2 Anaesthesiology, Tan Tock Seng Hospital, Singapore, SGP

**Keywords:** cerebral palsy, intestinal obstruction, intra-abdominal pressure, abdominal compartment syndrome, abdominal pain, constipation

## Abstract

The majority of patients with constipation can often be treated conservatively with laxatives, suppositories, or enemas in mild cases. However, endoscopic decompression or surgical intervention may be required in some instances. Abdominal compartment syndrome as a result of constipation is rarely seen in the literature. We report a case of faecal impaction, which led to abdominal compartment syndrome in an adult patient with cerebral palsy. With increasing life expectancy, such cases may be increasingly encountered in the adult population. Severe complications of constipation should not be overlooked, especially in this at-risk population. Early recognition of abdominal compartment syndrome is key in its management.

## Introduction

Constipation is a commonly encountered clinical problem with a global prevalence estimated to be about 18.9% in a systematic review [1]. In individuals with intellectual and developmental disabilities, it is estimated to have a higher prevalence of about 40% [2]. In daily practice, most cases of constipation are commonly treated with dietary modifications and medical therapy [3]. Severe constipation can be an intra-luminal cause of raised intra-abdominal pressure and potentially lead to an abdominal compartment syndrome (ACS). Abdominal compartment syndrome has been associated with poor clinical outcomes [4]. It is vital to recognize this in order to institute timely and effective treatment.

## Case presentation

A 60-year-old gentleman presented to the Emergency Department via ambulance for severe abdominal pain. He had a known history of dystonic cerebral palsy with intact cognition, chronic constipation and cervical spondylosis. Progressively worsening cervical spondylosis rendered him wheelchair-bound and he required assistance with activities of daily living (ADLs) over the last 10 years. He was not on any chronic medications. During these few years, he was cared for by his wife at home. He was subsequently lost to follow-up in the healthcare system, and had not had any endoscopic evaluation before. 

On arrival in the Emergency Department, he was in shock. He was afebrile with a temperature of 36.0 degrees celsius. He had a blood pressure of 65/43mmHg, heart rate of 113 beats per minute, respiratory rate of 32 breaths per minute and saturations of 95% on room air. History from the family revealed that he had been obstipated for two weeks. He subsequently experienced multiple episodes of overflow diarrhea, passing small amounts each time. He complained of worsening severe abdominal pain and was noted to be lethargic, prompting the family to seek medical attention. His abdomen was distended and tense, with absent bowel sounds.

**Figure 1:**
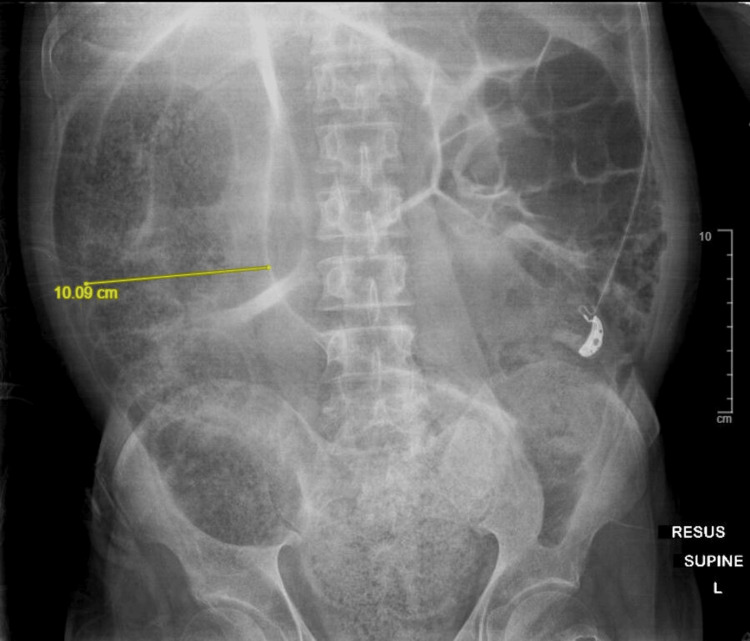
Abdominal X-ray showing faecal loading and dilated large bowel loops

An initial arterial blood gas showed metabolic acidosis with pH of 7.22, partial pressure of carbon dioxide (pCO2) 30mmHg, partial pressure of oxygen (pO2) 88mmHg, bicarbonate 12mmol/L, base excess of -15mmol/L and a lactate of 4.94mmol/L. Formal blood tests showed a creatinine level of 120umol/L (60-105umol/L), haemoglobin of 19.9 g/dL (13.6-16.6 g/dL), as well as a raised white blood cell count of 25.6x10^9/L (4.0-9.6 x 10^9/L) with 85.2% neutrophilia. There was a raised potassium at 5.8mmol/L (3.5-4.5mmol/L), although electrocardiogram showed sinus tachycardia with no tall T waves or ST-T segment changes. A total of 4 liters of intravenous (IV) Plasma-Lyte A was administered during the initial resuscitation while in the emergency department. Despite this, urine output monitored via an indwelling catheter (IDC) remained low. Intravenous antibiotics were commenced to cover empirically for an intra-abdominal cause of sepsis. Medical therapy for hyperkalemia was also administered. Abdominal X-ray (AXR) showed significant large bowel dilatation of at least 10cm (Figure 1).

**Figure 2:**
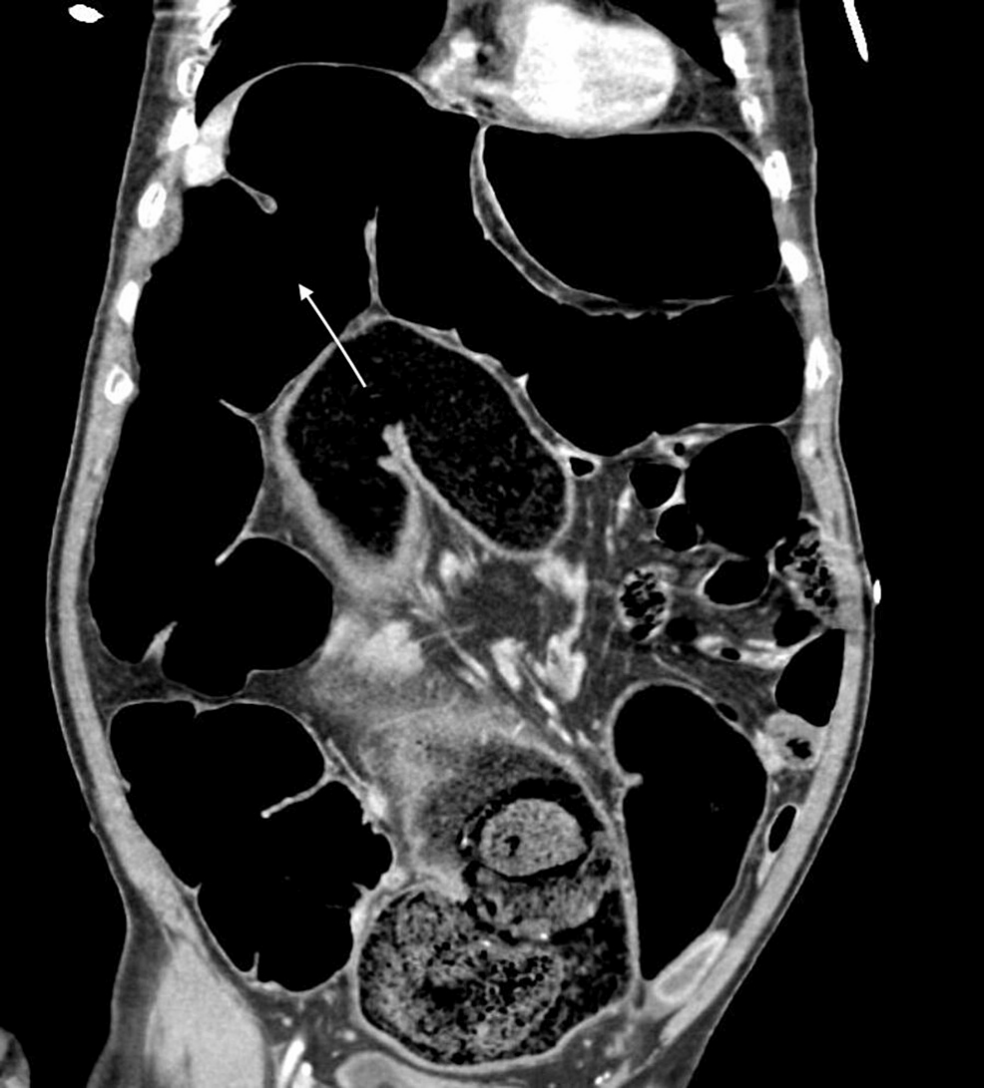
CT image showing dilated large bowel loops

An initial chest X-ray did not show any air under diaphragm. Initial concerns were that of a sigmoid volvulus, or acute abdomen secondary to intestinal obstruction and bowel perforation. A General Surgery (GS) consult was sought urgently and additional imaging was obtained once his hemodynamics stabilized. An urgent Computed Tomography (CT) scan showed dilatation of the colon with diameter up to 8.3cm, with a large amount of feces noted in the rectum and sigmoid colon (Figure 2). There was no definite transitional point and no reported pneumoperitoneum, but extrinsic compression of the inferior vena cava (IVC) was noted on the CT scan. Upon return from CT imaging, the patient was noted to have worsening tachypnoea ranging around 40-50 breaths per minute with borderline oxygen saturations at 93% on room air. He was subsequently placed on 2 liters of oxygen via nasal cannula. A repeat arterial blood gas was also done showing pH 7.19 pO2 86mmHg, pCO2 31mmHg, bicarbonate 12mmol/L, base excess -16mmol/L and lactate 5.79mmol/L.

A bedside manual digital evacuation and flatus tube insertion were performed by the GS team. An arterial line for closer hemodynamic monitoring and central venous access was obtained for the patient. Despite fluid resuscitation, arterial blood pressures continued to downtrend to as low as 78/67 mmHg. He was subsequently started on IV noradrenaline infusion at 0.2mcg/kg/min in view of persistent hypotension. In view of his worsening clinical status and respiratory effort, a decision was made for intubation and transfer to the Surgical Intensive Care Unit (SICU) for further resuscitation and plans for surgical decompression by the surgical team once stabilised.

**Figure 3:**
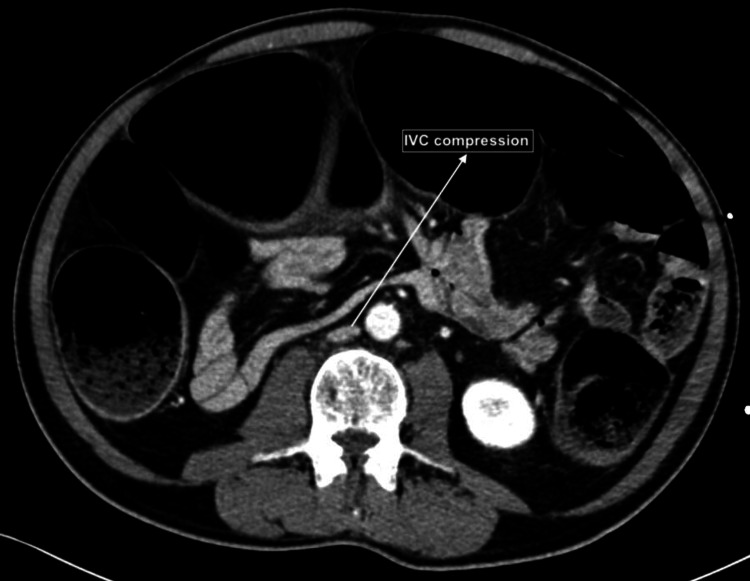
CT image of the abdomen in an axial cut showing compressed inferior vena cava (IVC)

On arrival to SICU, he was found to be persistently hypotensive with rising vasopressor requirements. Noradrenaline infusion was titrated to 0.5mcg/kg/min and vasopressin was started at 1.2units/hour. He was also noted to have high peak airway pressures between 41-49 cmH2O. His initial intra-abdominal pressure (IAP) measured via a Foley’s catheter was 49mmHg (0-10mmHg). This, coupled with the presence of vascular compression on CT scan (Figure 3) and acute kidney injury led to a diagnosis of abdominal compartment syndrome.

A surgical re-consult was urgently sought in the SICU for consideration of emergent surgical management. As this was being arranged, the patient rapidly deteriorated, with worsening hypotension despite fluid resuscitation and vasopressor support. He subsequently suffered a cardiovascular collapse with pulseless electrical activity (PEA) within the first hour of arrival to the SICU. Resuscitation was commenced as per the Acute Cardiac Life Support (ACLS) protocol with a return of spontaneous circulation (ROSC) initially. A bedside decompressive sigmoidoscopy was organized as he was not fit for transport due to severe hypotension requiring adrenaline 1mcg/kg/min, noradrenaline 1mcg/kg/min and vasopressin 2.4units/hr. Unfortunately, the patient suffered a further five episodes of PEA cardiovascular collapse with short and unsustained periods of ROSC in between.

Despite many rounds of resuscitation, the patient had persistently worsening IAP, acidosis and recurrent cardiovascular collapses. These rendered procedural or surgical interventions prohibitive. A decision was eventually made to hold off further resuscitation, in view of medical futility and the patient's premorbid status. Extensive discussions were held throughout the resuscitative process and this was also in line with the patient’s and family’s wishes. The patient eventually demised surrounded by his family.

## Discussion

In the case above, the patient was found to have raised intra-abdominal pressures and an eventual diagnosis of abdominal compartment syndrome. Although there is no strict IAP threshold, a sustained IAP of >/= 12mmHg is usually defined as intra-abdominal hypertension. Abdominal compartment syndrome is defined when IAP exceeds 20mmHg with associated new-onset organ dysfunction [5]. While there are various methods to measure intra-abdominal pressure, measurement of bladder pressure (intravesical pressure) is the standard method of measuring intra-abdominal pressure [6].

Our patient presented with ACS and shock resulting from constipation, which is a rare complication of constipation. Although ACS is more often described in patients with abdominal surgery, trauma or burns, there are a few case reports of ACS due to constipation. Birkhahn et al. [7] describe a case of ACS in a child with congenital megacolon. Flageole et al. describe an 11-year-old boy with chronic constipation and ACS [8]. In the adult population, there is very limited literature on ACS related to constipation in adults. A case report by Kongkatong et al. [9] describes an adult patient with chronic constipation on long-term clozapine with a likely diagnosis of ACS. Despite undergoing urgent decompressive laparotomy, the patient suffered a cardiac arrest on table and subsequently demised. In another report, Starnes et al. describe a case of a 50-year-old man with cerebral palsy and repeated hospital admissions with constipation. He presented to the emergency department in a state of cardiac arrest. The patient eventually demised and this was retrospectively attributed to abdominal compartment syndrome from severe constipation [10].

**Figure 4:**
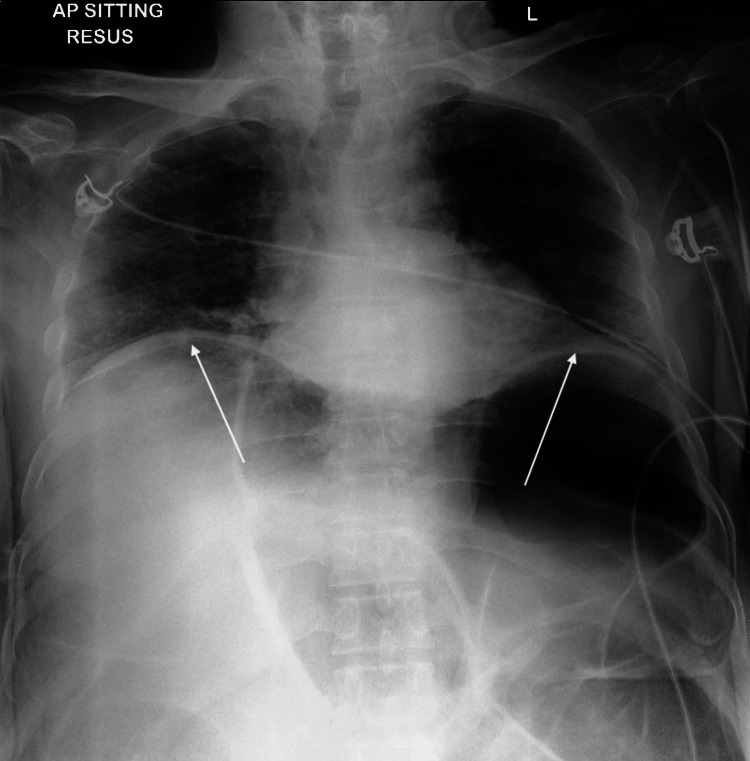
Decreased lung volume and cephalad displacement of diaphragm on chest X-ray

The management of ACS is reliant on early recognition of the syndrome itself. Patients often present with hypotension, tachycardia, abdominal pain and a significantly distended abdomen. Radiological or CT findings of vascular compression, such as IVC compression, are suggestive of ACS [11]. In this patient described above, there were other initial clues suggestive of ACS even prior to IAP measurement. These include clinical signs such as anuria, and radiological findings such as cephalad displacement of the diaphragm and decreased lung volumes seen on X-ray (Figure 4). 

In addition, there was reduced pulmonary compliance and high airway pressures during ventilation.

Initial management of ACS includes supportive management [12] such as ventilatory support with paralysis. Additionally, intra-luminal causes of ACS should be first addressed by the bedside by employing nasogastric and rectal decompression. The patient’s fluid balance should be closely monitored; with volume loading to ensure adequate venous return while avoiding over-resuscitation to minimize third spacing. Similarly, sedation and pain management can also improve abdominal wall compliance. However, definitive management often lies in emergent surgical decompression.

Constipation is often treated as a benign condition that can be treated with medications or a procedure such as a decompressive scope. Our case adds to the limited literature and highlights a potentially dangerous complication of untreated constipation, where ACS can occur and patients may enter a state of obstructive shock. Severe complications of constipation should especially be anticipated in at-risk populations such as those with cerebral palsy, where complications like volvulus and perforation have been known to occur [13]. Although ACS secondary to constipation is rare, early recognition of the syndrome is key in its management. On reflection, earlier recognition and surgical decompression should have been considered promptly in a rapidly deteriorating patient with ACS. It was unfortunate that the patient had multiple episodes of cardiac arrest. This rendered him unsafe for any transport or surgical procedures at that stage, and eventually led to his demise. 

## Conclusions

Abdominal compartment syndrome secondary to constipation is not commonly encountered. However, severe cases of chronic constipation can result in ACS, which physicians should consider especially in at-risk populations such as those with cerebral palsy. Evidence of end organ injury with objectively raised IAP enables the physician to make the above diagnosis of ACS. Our case is an unfortunate one whereby the patient did not survive. Early suspicion and diagnosis would have allowed for appropriate supportive and definitive management to be instituted.
